# Hypnotism

**Published:** 1860-04

**Authors:** O. D. Palmer

**Affiliations:** Zelienople, Pa.


### ARTICLE XV.

*Hypnotism.* Translated by O. D. Palmer, M. D., Zelienople, Pa.

> Hoc Gallicæ consurtudinis—

> Rumoribus aque auditionibus permoti,

> Summis sæpe rebus consilia ineunt.

>

> *Cæsar's Com. lib. 4.*

An enthusiasm amounting almost to ecstacy, has been recently produced in the scientific world of Paris, by the supposed discovery of a new method of effecting anesthesia. This novel method, which bears the appellation of *hypnotism,* is made to consist in a species of extemporaneous strabismus, caused by conveying the axis of the eyes to a point a few inches from the root of the nose. In this operation, certain muscles, the *recti superioris,* and the *levatores palpebrarum,* are in a state of forced contraction, and the continuance of this action, during a period varying from three to fifteen minutes, superinduces a state of the sensorium, identical with catalepsy, or at least analogous to it, called a hypnotic state. The extracts which follow, translated from recent copies of the *Gazette Hebdomadiare,* will give some intimations of the discovery, introduction, reception, and success, of this marvellous agent, now monopolizing the attention of that metropolis of science and civilization, Paris.

A young provincial surgeon, Dr. Azam, adjunct professor in a school at Bordeaux, brought to Paris this singular method, the fruit of prolonged studies and numerous experiments, patiently instituted, during a long period. Eighteen months since, he had occasion to attend a young hysterical patient in spontaneous catalepsy. He observed in her exceedingly curious facts, which it does not come within our province to relate here. A professor in the Academy of Sciences, Dr. Bazin, being instructed by his experiments, advised Dr. Azam to examine an English work, published in 1842, by M. Braid, and in which is found indicated a means of producing artificial catalepsy, and anesthesia. Dr. Azam, having procured the work, of which there is given an analysis by Carpenter, in the Cyclopedia of Todd & Bowman, (article *Sleep,*) he instituted upon this young cataleptic, and nearly thirty others, numerous experiments. He ascertained the greater part of Braid's assertions to be substantially correct, among others, that catalepsy and anesthesia could be procured at will, by proceeding in the following manner:

The subject is sitting or lying in a convenient position, the operator puts before his eyes, at some three inches distance, and generally within the point of distinct vision, a bright body, on which the eyes are to be directed, and fixed continuously; the body should be so placed, that the eyes are directed upward and inward, by the firm contraction of the proper muscles, causing convergent strabismus. Hardly has this fatiguing attitude been preserved in for two or three minutes, till we see the pupils contract, and then dilate, the palpebra oscillate rapidly, and then fall down, and immediately the subject is asleep. Two symptoms attend this state, catalepsy precisely as described in the class books, and anesthesia, enduring from three to fifteen minutes, complete or incomplete, but which generally permits pinching, pricking, and tickling, without the least trace of sensibility, or without modifying in the least the cataleptic state. This state of anesthesia is usually succeeded by the opposite state, of hyperæsthesia, in which we see the ordinary senses, the sensation of temperature and of muscular activity, attains a degree of more than usual impressibility; at any moment of the experiment, the symptoms can be made to suddenly cease, by frictions and sufflations of cold air on the eyelids, in a similar manner to what has been seen in the researches of Dr. Paul on catalepsy. The subjects, returned to their normal state, preserve no remembrance of what has passed during the preceding moments.

The gentlemen engaged with this subjeet specially, Drs. Azam, Broca, Fallen, and Velpeau, merit at least an examination, and they should not be assailed under what pretext soever, with incredulity, or even obstinate doubt. It has been a long time a reproach to the learned, their proud disdain for the extraordinary. We are in an epoch, in which all that is announced under a serious mien, and proceeds scientifically, merits examination; we live in a time, in short, when it would be unreasonable to turn away the eyes, merely because what is shown us is simply improbable and marvellous. Furthermore, the better way, and indeed the only, to judge anything, consists in at first looking it in the face, and this is what has been done by the grave men we have cited; this is what we have undertaken to do ourself. We will merely add, that Dr. Azam has arrived in Paris, fully persuaded that surgery was in possession of a new anesthetic. The readers may judge from the following, of the value of this impression.

We hasten to lay before our readers a case by an ancient colleague, a distinguished provincial surgeon, Dr. Gruri-neau, adjunct professor in the secondary school at Poictiers.

In the case that follows, we have not to do with an impressible woman, hysterical, and predisposed, by a nervous fantastic system, to extatic manifestations, more or less poetic and marvellous. The subject was a peasant, a little nervous, lymphatic, exhausted, and anything but a stoic.

*Case.*—George Jarry, aged thirty-four years, from the village of Mortimer, had been treated for several months in a hospital, for a white swelling of the left knee. So painful was this knee, that the least motion caused the patient to cry out. He had given his consent that the leg should be amputated at the thigh.

I operated in the presence of several distinguished surgeons. One of them held a spatula within about three inches of the root of the nose of the patient, whilst lying in a horizontal position. Strabismus convergent upward, was promptly produced.

Five minutes had elapsed, since his eyes had been fixed upon the spatula; I raised the left arm up, and let it go; immediately it fell. Then there was no catalepsy. The patient said we would not be able to put him to sleep by this process. I immediately recommended the greatest silence in the apartment, where numerous parties had begun conversations: I spoke no further to the patient, who eyed the spatula with perseverance. After five minutes of the most profound silence I performed amputation of the inferior part of the thigh, by the double flap operation. During this operation, which lasted a minute and a half, the patient did not make the least plaint or motion; I now spoke to him, and inquired how he was. He said in answer, he thought himself in paradise, and seizing my hand carried it to his lips. During the operation his eyes were affected by a twitching movement. They had the appearance of searching for the spatula. A student, pinching his thigh a couple of minutes before the operation, asked if it gave pain, "O, I feel it a little," said he. After the operation, Jarry said "he knew the time the leg was cut off, for at that period, they asked him if he had any pain." Now it was two minutes after this interrogation, that the operation commenced, and during all the time of this, his visage offered not the least spasm or contraction. All this time the eyes of Jarry seemed to search for the spatula.

It was quite evident to the assistants, that the patient did not experience pain, as he did not make the least plaint, whilst previously he cried out on the least motion of the affected limb.

---

When Dr. Azam reads the above, he will, without doubt, experience a lively satisfaction; he will find in it encouragement to pursue it, without too much pre-occupation with certain malevolent checks. We wish, moreover, he may induce anæsthesia of his auditory nerves, until the uproar of skeptics shall be appeased. As for our own part, without abdication of any doubts, or any philosophic prudence, we continue to observe and to reflect.

AR. VERNEIUL.

---

The following case appears to have occurred in Paris.

*Case.*—Woman aged twenty-four years—extensive burn of back and right limbs—abscess of the verge of anus, voluminous, and very painful—weakened by pain, and otherwise pusillanimous, greatly fears incision—was told she would be put to sleep—a copper cylinder, (Brick's lunette,) placed three inches before root of nose—patient to see this object, was obliged to squint strongly; pupils immediately much contracted, pulse rapid previous to experiment, first a little accelerated, then becoming feeble, slow; two minutes are passed, pupils dilate, left arm raised vertically from the bed; remains immovable in that position; toward fourth minute, answers are slow, laborious, but perfectly sensible; respiration slightly affected. At the end of five minutes M. Follen pricks the skin of the left arm, in vertical position; no movement; pricks again, giving rise to a drop of blood ; passed equally unperceived, right arm placed in attitude same as left arm ; laid bare seat of abscess ; patient permits all, quietly saying she fears to incur some injury from us. In short, seven minutes after the *debut*, Dr. Follen made a large opening into the abscess, which gives issue to a large quantity of fœtid pus. A light cry, lasting less than a second, was the only sign given by patient of feeling ; otherwise not the least twinge in the muscles of the face, or limbs ; arms have constantly maintained the attitude first given them.

Two minutes more, the position ever the same ; eyes ever wide open ; a little injected ; the visage impassible, the pulse as before the experiment ; the respiration perfectly free, the subject always insensible. We raised the right foot, it remained suspended in the air ; the cataleptic state of superior limbs present.

Dr. Broka removed the bright body, which had constantly been kept before the eyes : he used friction over the eye-lids, and a stream of cold air. The patient made some little motion. She was asked if anything had happened to her. She answered she knew of nothing. The three limbs remain still in the attitude given them. Pricking again upon the left arm is not perceived. Eighteen minutes after the commencement of the experiment ; twelve minutes after the operation, friction again, sufflation again on palpebra ; sudden awaking ; the members in catalepsy fall all at once. The patient rubs her eyes, and resumes her consciousness. She remembers nothing, and is astonished at having been operated upon. Her state is comparable, as far as a certain point, to that of an individual who has come out of an ordinary anæsthetic sleep. Always the waking is more sudden ; without agitation, and without loquacity. The anæsthesia, interrupted by the provoked waking, has lasted at least fifteen minutes.

---

We understand that Azam has succeeded in anæsthizing a young girl. under the eyes of Prof. Trousseau, very promptly, and all leads to the belief that these experiments will be multiplied.

We ought merely to anticipate, that some subjects show themselves altogether refractory to *hypnotism.*

We wish, in conclusion, to forewarn our brethren, against too sudden an enthusiasm, as well as against an ultra skepticism. We should study the question coolly, calmly, and patiently. The promoters of this discovery, as well as those who seek to propagate it, do not wish to deceive, or to be deceived. [*Ed. Gaz. Heb.*

